# Geriatric oncology in Mexico

**DOI:** 10.3332/ecancer.2020.1102

**Published:** 2020-09-15

**Authors:** Carolina Gomez-Moreno, Haydeé Verduzco-Aguirre, Enrique Soto-Perez-de-Celis

**Affiliations:** 1 Department of Geriatrics, Instituto Nacional de Ciencias Médicas y Nutrición Salvador Zubirán, Mexico City, 14080, Mexico; 2 Department of Hemato-Oncology, Instituto Nacional de Ciencias Médicas y Nutrición Salvador Zubirán, Mexico City, 14080, Mexico

**Keywords:** geriatrics, Mexico, developing countries, oncology, clinical, capacity building

## Abstract

Mexico is an upper-middle income country located in North America, with an increasing life expectancy and a growing population of older adults. Due to population ageing and lifestyle changes, the number of cancer cases amongst older individuals is expected to increase in the upcoming decades, representing a challenge to the healthcare system. The challenges for implementing geriatric oncology care in Mexico include a fragmented healthcare system, as well as the lack of geriatric training amongst healthcare professionals. In this mini-review, we present an overview of the epidemiology of cancer amongst older adults in Mexico, the Mexican healthcare system and ongoing initiatives for the development of geriatric oncology programmes in the country. In addition, we highlight the priorities for future developments in the field of cancer and ageing in Mexico, with a focus on the various stakeholders involved.

Mexico is a middle-income country located in North America. With a territory of over 2 million square kilometres and a population of 130 million people, Mexico is the 13th largest and 10th most populous country in the world [1]. As in many developing countries, life expectancy in Mexico has continuously improved and is currently 74.9 years at birth and 21 years for adults aged between 60 and 64 years [1, 2]. This has led to a rising proportion of older adults, with approximately 7.2% of Mexicans aged ≥65 years in 2018 [1]. Although the proportion of older adults is still low compared with high-income nations, projections show that 17% of Mexicans will be aged ≥65 by 2050, with most of these demographic changes taking place in the more prosperous northern regions and urban areas around Mexico City [1, 3].

Concurrent economic growth has also brought along more sedentary lifestyles, unhealthy dietary habits, smoking, alcohol consumption and other exposures, leading to an increase in non-communicable diseases (NCDs) such as cancer [4]. Besides this increasing incidence, there is a large cancer-related mortality burden, even when compared to other areas in the world with higher incidence rates [4]. This rising burden of cancer amongst older adults impacts healthcare systems in terms of both the increasing number of people in need of care and the increasing level of complexity in cancer management, underlining the need for initiatives aimed at providing high-quality care for older adults with cancer [5].

In this mini-review, we highlight both the challenges for implementing geriatric oncology in Mexico and the current initiatives undertaken throughout the country. In addition, we provide a set of recommendations for future actions in order to move geriatric oncology forward.

## Cancer care and the Mexican healthcare system

**Figure 1.**
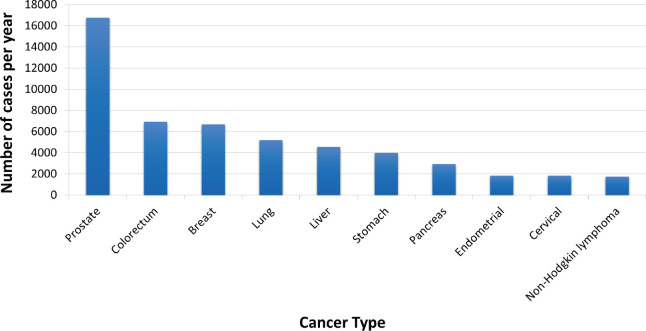
Top ten most common malignancies amongst adults aged 65 years and older in Mexico, 2018 (absolute number of new cases). Source: Globocan 2018 [4].

Cancer is an important source of morbidity and use of health resources in Mexico. In 2018, almost 200,000 new cancer cases were diagnosed, of which 69,000 occurred in older individuals, and over 80,000 cancer-related deaths were registered, with more than half (43,000) occurring amongst older adults [6]. The top ten most frequent cancers in Mexico amongst older adults (excluding non-melanoma skin cancer) are shown in Figure 1 [6].

**Figure 2.**
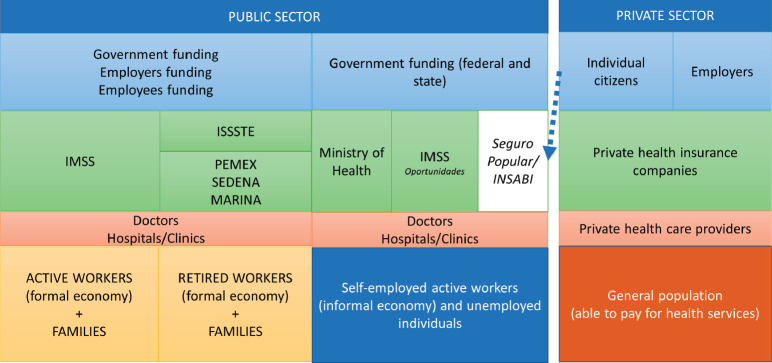
Schematic outline of the Mexican healthcare system. Adapted from Gómez-Dantes et al [7].

Unfortunately, the Mexican healthcare system is ill-prepared to face the ageing population and the associated increase in NCDs due to numerous factors including system fragmentation, biased allocation of resources, underinvestment in equipment and infrastructure and inequities in access to care [7]. Generally speaking, the Mexican healthcare system is composed of two sectors (Figure 2), both of which offer cancer care: public and private [8]. The public sector comprises institutions which provide social security to formally employed sectors of the population, as well as institutions which provide healthcare services to the self-employed or unemployed, mainly through government funds assigned to the *Seguro Popular* public insurance system [9, 10]. The private sector provides healthcare services to those who have enough resources to pay for healthcare themselves or those with private insurance (approximately 5% of the population) and is mainly financed by its users [9].

Healthcare system fragmentation is a challenge for cancer care since each institution has its own set of covered interventions, which vary greatly. In contrast with other countries with unified single-payer public healthcare systems, such as the United Kingdom, Mexico has a variety of subsystems, with different payers, benefits and accessibility, leading to stark inequalities in access and outcomes [4, 7, 8]. Patients attempting to access the system may be unable to do so due to the lack of availability of oncology services at their assigned healthcare institution locally, and they may need to travel long distances to obtain care. Furthermore, the lack of interaction between healthcare systems represents a challenge for the creation of programmes aimed at providing geriatric oncology care since some specialists (geriatricians, physical therapy, nutrition and so on) may not be available within the patient’s institution.

## Geriatric training in Mexico

Another barrier for the establishment of geriatric oncology programmes is that Mexico’s healthcare professionals receive insufficient geriatric training. Geriatric training is not mandatory in medical school or general residency training, limiting the exposure of trainees to geriatrics and making it harder for them to develop an interest in the field [11]. Core geriatric competencies are insufficiently taught throughout medical and allied health training, leading to ageist attitudes and perceptions. A study amongst Mexican physicians in training demonstrated a high prevalence of mostly negative perceptions of ageing, exemplified by the use of negative terms to describe older adults [12].

To make matters worse, opportunities for specialised geriatric training in Mexico are limited, and geriatric oncology is not included in geriatrics training programmes. In 2010, only ten universities offered specialisation programmes in geriatrics, with most of these located in Mexico City, which may contribute to discourage the pursuit of a career in geriatrics for physicians in other regions of the country [11].

In order to encourage physicians to become geriatricians, the Ministry of Health shortened geriatric fellowship training from 6 to 4 years, leading to an encouraging increase in the number of available fellowship positions from 86 in 2016 to 132 in 2019 [13]. Despite this increase, there is still a worrisome lack of geriatricians, with only 615 certified geriatricians for a population of 9.1 million older adults (14,822 older adults per geriatrician) [1, 14].

## Current geriatric oncology initiatives

Despite being an emerging area of cancer care, Mexican geriatric oncology has grown during the past 5 years. The first multidisciplinary geriatric oncology clinic was created at *Instituto Nacional de Ciencias Médicas y Nutrición Salvador Zubirán* (INCMNSZ) in 2015 [15]. This clinic, which is a part of the public healthcare sector, currently employs three attending physicians (two geriatricians and one medical oncologist), along with experts in nutrition, rehabilitation/physical therapy, palliative care and social work in older adults. The clinic employs a consultative model, in which patients are comanaged together with their primary treating oncologist or haematologist, and recommendations regarding treatment and supportive care are issued, with approximately ten new patients aged 65 and older seen on a weekly basis. Geriatric oncologists also attend multidisciplinary tumour boards [15]. Recently, the clinic reported a high agreement between geriatric oncology recommendations and final treatment plans by the treating oncologist, which was improved in cases, where the geriatric assessment was mentioned by the oncologists’ notes [16]. As an additional benefit, residents and fellows from the geriatric, medical oncology departments and other institutions in the country have rotations in the geriatric oncology clinic during their training.

The communication of geriatric oncology research and continuing medical education has been undertaken by several institutions and national associations. The Mexican Society of Oncology (SMeO), for example, included a geriatric oncology track in its 2017 Annual Meeting. In 2018, INCMNSZ’s International Geriatrics Meeting was focused on geriatric oncology and geriatric haematology under the auspices of the International Society of Geriatric Oncology, the SMeO and the National College of Geriatric Medicine. At this meeting, physicians from Mexico and Latin America were given a platform to discuss their research in geriatric oncology and establish cooperative efforts with international experts.

Despite the advances in recent years, research in geriatric oncology in Mexico is still limited. Most publications in the field come from the National Institutes of Health or private hospitals located in Mexico City, sometimes in collaboration with international authors. The aforementioned geriatric oncology clinic at INCMNSZ has published its experience in the implementation and feasibility of the clinic, as well as other original research projects, with a focus on the use of mobile health in geriatric oncology [16–18]. This has been made possible partly through research grants from the Conquer Cancer Foundation of the American Society of Clinical Oncology, the Global Cancer Institute and the *Miguel Aleman* Foundation. In addition, awareness of the particularities of cancer management in older adults has grown, and geriatric principles have been included in the national guidelines, such as the 2019 Mexican Consensus on Breast Cancer Diagnosis and Treatment [19].

## Future perspectives and needs

Although Mexico is still a relatively ‘young’ country, the first half of the 21st century will bring accelerated population ageing, which will translate into a high proportion of older adults with cancer. Therefore, all sectors of the Mexican healthcare system will need to prepare in order to increase their geriatric competencies and provide high-quality care for this increasingly complex patient population. The federal government has announced that providing healthcare for older adults will be one of its priorities and has created programmes aimed at supporting older adults, including the ‘Pension for the Well-being of Older Persons’ managed by the newly created Ministry of Well-being. It is yet to be seen if other changes to the structure of the healthcare system, such as the implementation of the ‘National Institute of Health for Well-being’ (INSABI) which is designed to provide universal healthcare (including treatment for all cancer types), will lead to an improvement in healthcare system coordination.

Regardless of the future changes to the structure of the healthcare system, there is a need to increase the geriatric competencies of the entire healthcare workforce, including allied health professionals, and to combat ageism amongst healthcare workers. In addition, there is a need to foster capacity building through the creation of national centres of excellence in geriatric oncology, which can design and test clinical care models, train specialists and conduct research into cancer and ageing, with a particular focus on reporting the impact of geriatric oncology care on patient outcomes. Geriatric oncology principles should be embedded into national cancer care guidelines, following the example of the aforementioned breast cancer consensus, and cancer care for older adults should be prioritised in future national plans and universal health coverage schemes. Finally, cooperation between national and international organisations should be fostered to increase funding opportunities, improve clinical care and create international training initiatives. These recommendations are shown in Table 1.

## Conclusions

In summary, whilst Mexican geriatric oncology faces many challenges, it also has many opportunities for growth. The following years will be critical for increasing the preparedness of the healthcare system for the impending increase in the number of older adults with cancer, and an effort from all stakeholders will be required to achieve this goal.

## Conflicts of interest

The authors declare no financial conflicts of interest related to this manuscript.

## Authors' contributions

Conception and design: all authors.

Acquisition of data: all authors.

Data interpretation and analysis: all authors.

Drafting of manuscript: all authors.

Critical revision: all authors

## Funding declaration

No funding was received for the writing of this manuscript.

## Figures and Tables

**Table 1.** Selected priorities for the development of geriatric oncology in Mexico.

Priority	Involved stakeholders	Recommendations
Improving education and training	Universities Government bodies Medical societies	Integrating geriatric principles into medical school education. Integrating geriatric oncology principles in the training of oncology professionals, including allied health workers. Organise workshops and continuous medical education activities aimed at teaching geriatric principles and core competencies.
Provide structural support for the establishment of geriatric oncology	Healthcare system Government bodies Universities	Provide integrated universal health coverage for older adults with cancer. Develop and foster multidisciplinary geriatric oncology models of care. Promote the creation of centres of excellence in geriatric oncology. Create additional fellowship positions in geriatrics and specialisation programmes in geriatric oncology.
Foster the dissemination of geriatric oncology principles and the generation of new knowledge	Healthcare system Government bodies Medical societies Universities	Include geriatric principles in cancer clinical care guidelines. Include geriatric principles in national cancer plans. Develop links between the national and international organisations to foster research and training. Develop funding mechanisms for cancer and ageing research.
